# Titanium dioxide nanoparticles induce oxidative stress and DNA-adduct formation but not DNA-breakage in human lung cells

**DOI:** 10.1186/1743-8977-6-17

**Published:** 2009-06-21

**Authors:** Kunal Bhattacharya, Maria Davoren, Jens Boertz, Roel PF Schins, Eik Hoffmann, Elke Dopp

**Affiliations:** 1 Institut für Hygiene und Arbeitsmedizin, Universität Duisburg-Essen, Germany; 2 Radiation and Environmental Science Centre, FOCAS Institute, Dublin Institute of Technology, Dublin, Ireland; 3 Institut für Umweltanalytik, Universität Duisburg-Essen, Essen, Germany; 4 Institut für Umweltmedizinische Forschung (IUF) an der Heinrich-Heine University Düsseldorf, Germany; 5 European Molecular Biology Laboratory (EMBL), Heidelberg, Germany

## Abstract

Titanium dioxide (TiO_2_), also known as titanium (IV) oxide or anatase, is the naturally occurring oxide of titanium. It is also one of the most commercially used form. To date, no parameter has been set for the average ambient air concentration of TiO_2 _nanoparticles (NP) by any regulatory agency. Previously conducted studies had established these nanoparticles to be mainly non-cyto- and -genotoxic, although they had been found to generate free radicals both acellularly (specially through photocatalytic activity) and intracellularly. The present study determines the role of TiO_2_-NP (anatase, ∅ < 100 nm) using several parameters such as cyto- and genotoxicity, DNA-adduct formation and generation of free radicals following its uptake by human lung cells *in vitro*. For comparison, iron containing nanoparticles (hematite, Fe_2_O_3_, ∅ < 100 nm) were used. The results of this study showed that both types of NP were located in the cytosol near the nucleus. No particles were found inside the nucleus, in mitochondria or ribosomes. Human lung fibroblasts (IMR-90) were more sensitive regarding cyto- and genotoxic effects caused by the NP than human bronchial epithelial cells (BEAS-2B). In contrast to hematite NP, TiO_2_-NP did not induce DNA-breakage measured by the Comet-assay in both cell types. Generation of reactive oxygen species (ROS) was measured acellularly (without any photocatalytic activity) as well as intracellularly for both types of particles, however, the iron-containing NP needed special reducing conditions before pronounced radical generation. A high level of DNA adduct formation (8-OHdG) was observed in IMR-90 cells exposed to TiO_2_-NP, but not in cells exposed to hematite NP. Our study demonstrates different modes of action for TiO_2_- and Fe_2_O_3_-NP. Whereas TiO_2_-NP were able to generate elevated amounts of free radicals, which induced indirect genotoxicity mainly by DNA-adduct formation, Fe_2_O_3_-NP were clastogenic (induction of DNA-breakage) and required reducing conditions for radical formation.

## Background

Titanium dioxide (TiO_2_) has several isoforms of which anatase is the most commercially used type. To date, no parameter for the average ambient air concentration of TiO_2 _nanoparticles have been set by any regulatory agency. For the finer particles American conference of governmental industrial hygienists (ACGIH) has assigned TiO_2 _a threshold limit value of 10 mg/m^3 ^(total dust) as a time weighted average for a normal 8 h workday and a 40 h workweek [1]. Occupational exposure to TiO_2 _nanoparticles can occur during manufacturing/use of these particles as pigments for paints, varnishes, enamels, lacquers and paper coatings to impart whiteness, opacity and brightness [2]. Exposures can also occur from their use in cosmetics such as in sunscreens, dusting powder, ointments and from radioactive decontamination of the skin. The small size of the nanoparticles provides them with special properties such as high surface to volume ratio and high surface charge. The surface charge is one of the major physical properties of the nanoparticles that play a major role in their industrial application. It can only be measured indirectly through the zeta potential which is a function of the surface charge of the particle or any adsorbed layer at the interface and the nature and composition of the surrounding medium in which the particle is suspended. The zeta potential however, gives no information on the elemental composition of the surface [3].

Similar to TiO_2_-NP, hematite nanoparticles (Fe_2_O_3_-NP), which were used in the present study for comparison, have several industrial uses such as the magnetic layer of storage devices in computers and pigments for paints. Hematite is the mineral form of Iron(III) oxide (Fe_2_O_3_), has an iron content of about 70% and is one of the most important industrial used iron oxide beside magnetite (Fe_3_O_4_). Recently, Fe_2_O_3_-NP have been used for drug targeting cancer cells [4] and for labelling and tracking target cells using imaging techniques like magnetic resonance [5]. Exposure to hematite dust has been observed to cause siderosis in workers involved in iron mining, crushing, rolling industries and foundries and the regular end product users [6]. However, similar to TiO_2_-NP no ambient air threshold concentration limit has been set for these nanoparticles by any regulatory agency.

*In vivo *and *in vitro *toxicological studies confirm that for low solubility, low toxicity materials such as TiO_2_, carbon black and iron oxide, ultrafine particles are more toxic and inflammogenic than fine particles [reviewed in [7]]. In such studies the NPs generate reactive oxygen species (ROS) to a greater extent than larger particles leading to increased transcription of pro-inflammatory mediators via intracellular signaling pathways including calcium disturbances and oxidative stress [7] To date, only limited NP compositions and structures have been tested.

In the present study human lung fibroblasts (IMR-90) were exposed to TiO_2_- or Fe_2_O_3_-NP for analyzing their cyto- and genotoxic potential, their ability to generate reactive oxygen species and to form DNA-adducts. For these investigations additional studies regarding the physico-chemical properties of the particles were undertaken such as measurement of surface area (BET), particle size distribution, zeta potential, electron dispersive X-ray analysis (EDX) of surface chemistry, trace Fe(II) and Fe(III) elemental determination, and electron microscopy studies.

## Results

### Physico-chemical characterization of nanoparticles (NP)

EDX spectral analysis of the particle surface chemical composition revealed that the TiO_2 _nanoparticles contained 56% titanium (Ti), 41% oxygen (O) and 3% carbon (C) elements, while Fe_2_O_3_-NP were purely composed of 78.7% iron (Fe) and 21.3% oxygen (O).

**Figure 1:**
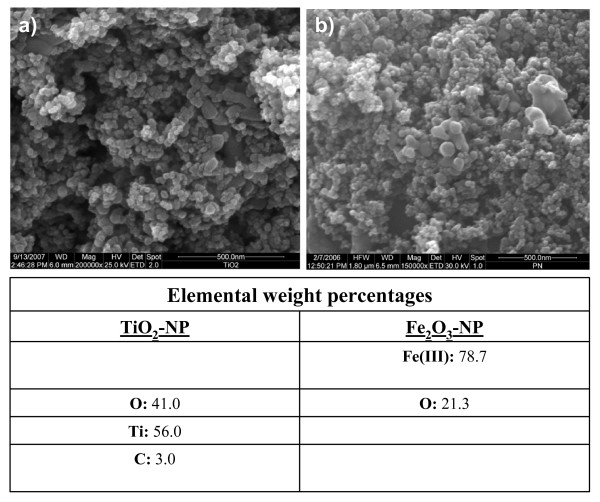
**Scanning electron microscopy images of (a) titanium dioxide and (b) hematite nanoparticles and EDX spectral analysis of particle surface composition**.

Zeta potential measurements revealed that TiO_2_-NP had a +48.8 mV charge and the average particle hydrodynamic diameter proved to be 91 nm. Fe_2_O_3_-NP demonstrated a zeta potential of -28.68 mV and a particle hydrodynamic diameter of ~50 nm. Morphologically, using Scanning Electron Microscopy (SEM) both the nanoparticles were found to be spherical in shape (Fig. 1).

### Electron microscopy studies

**Figure 2:**
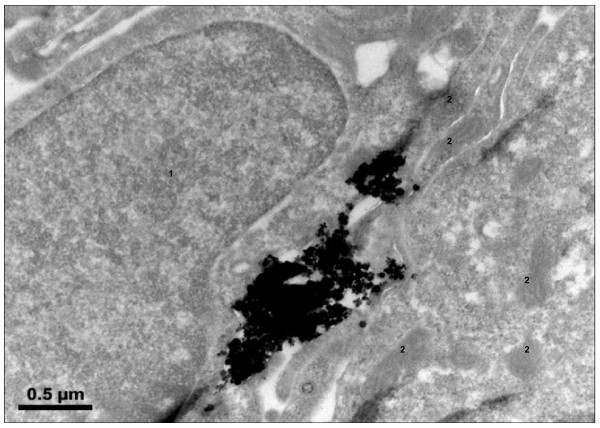
**BEAS-2B cell exposed to hematite-NP (10 μg/cm^2 ^for 48 h)**. Agglomerated and single particles were found near the nucleus (1), in the endoplasmic reticulum or lysosomes and surrounded by mitochondria (2). No particles were found within the nucleus or intracellular organelles such as mitochondria or ribosomes. Similarly, also the TiO_2_-NP were located in the cytosol (mainly in lysosomes) without entering the nucleus.

For investigation of uptake and translocation of NP, BEAS-2B cells (bronchial epithelial cells) were used. TiO_2_- as well as Fe_2_O_3_-NP were taken up by the cells by filopodia assisted engulfment of particle clusters or by micropinocytosis (single nanoparticles). Intracellularly, the NP clusters were located in vacuole-like structures. Single NP were found also in lysosomes of the bronchial epithelial cells. Within 48 h both types of NP were translocated to the peri-nuclear region of the cell, however, no particles were found to have penetrated the nucleus or mitochondria and ribosomes. Fig. 2 shows hematite NP near to the nucleus (1) and surrounded by mitochondria (2). The cell organelles were not affected after an exposure time of 48 h. Both types of NP showed a similar intracellular distribution and accumulation.

### Cytotoxicity and genotoxicity analyses

**Figure 3:**
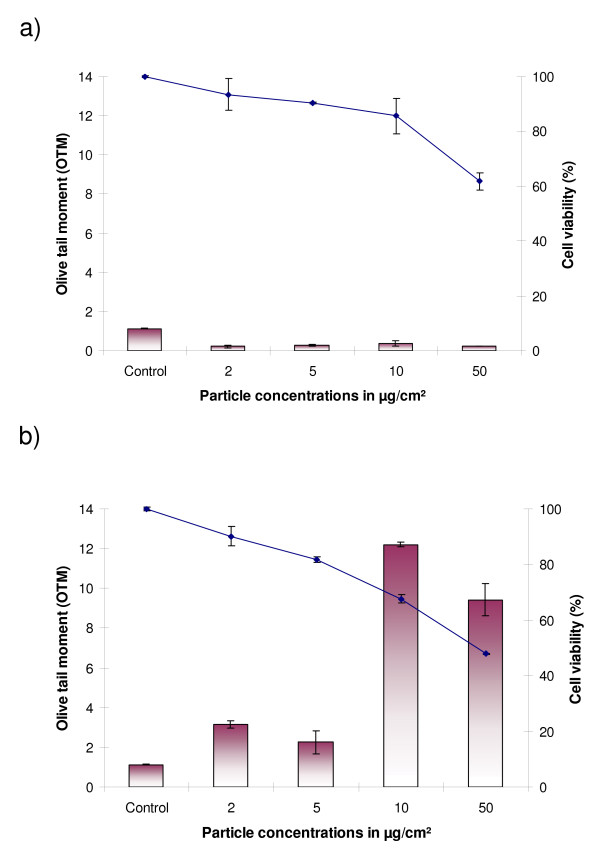
**IMR-90 cells exposed to titanium dioxide (a) and hematite (b) nanoparticles (exposure time: 24 h)**.

**Figure 4:**
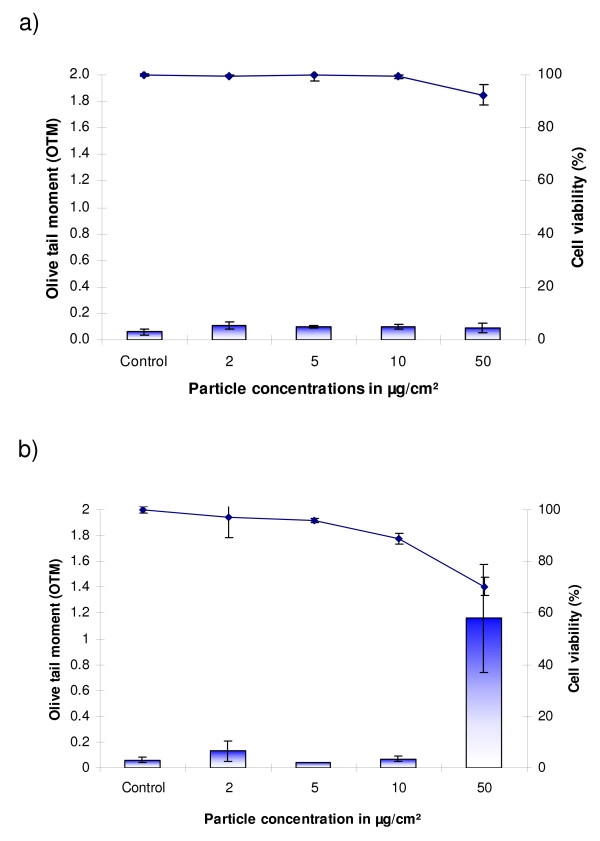
**BEAS-2B cells exposed to titanium dioxide (a) and hematite nanoparticles (b) for 24 h**.

Cytotoxicity and genotoxicity studies were carried out in both types of human lung cells: IMR 90 (human bronchial fibroblasts) and BEAS-2B cells. IMR-90 cells were more sensitive and showed stronger effects after particle exposure than the virus-transformed BEAS-2B cells (Figs. 3 and 4). Hematite NP induced a concentration-dependent loss of cell viability after 24 h exposure in both cell lines (p ≤ 0.05). TiO_2_-NP did not induce cytotoxic effects in BEAS-2B cells up to a tested concentration of 50 μg/cm^2 ^(Fig. 4), whereas significant cytotoxic effects were observed in IMR90-cells (Fig. 3).

TiO_2_-NP did not induce DNA-breakage measured by the Comet-assay in both human cell lines (Figs. 3 and 4). In contrast, exposure of cells to Fe_2_O_3_-NP induced significant DNA-damage. After 24 h exposure of IMR-90 cells, DNA-breakage was observed at concentrations of 10 (p ≤ 0.001) and 50 μg/cm^2 ^(p ≤ 0.01) and in BEAS-2B cells at 50 μg/cm^2 ^(p ≤ 0.01).

### Electron paramagnetic resonance (EPR)

**Figure 5:**
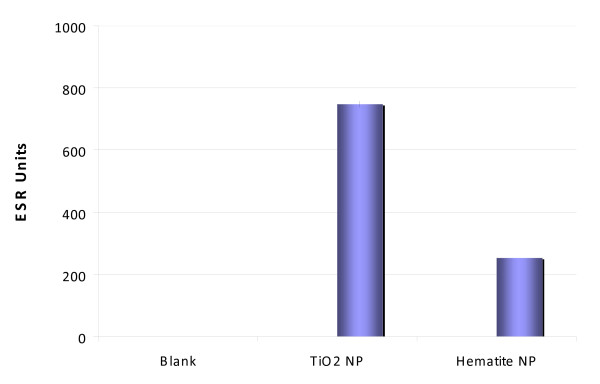
**Electron paramagnetic resonance measurement of acellular reactive oxygen species being generated by titanium dioxide and hematite nanoparticles**.

**Figure 6:**
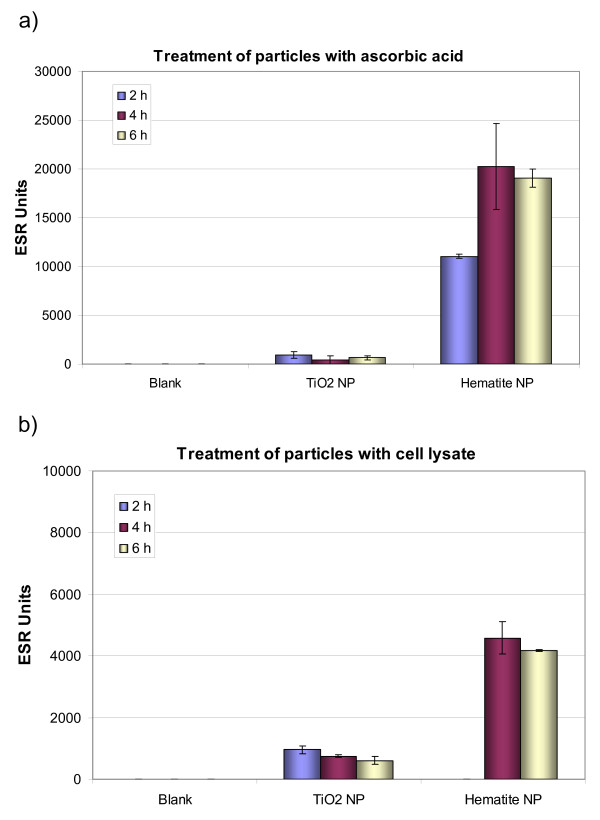
**Electron paramagnetic resonance measurement of acellular reactive oxygen species being generated by titanium dioxide and hematite nanoparticles a: treatment of particles with ascorbic acid and H_2_O_2_, b: treatment of particles with cell lysate and H_2_O_2_)**.

Both types of NP were analyzed regarding their potential to produce reactive oxygen species (ROS) in an acellular system. Interestingly, the measured ESR-units were lower for the iron-containing hematite NP than for TiO_2_-NP (Fig. 5). In a next step both types of NP were treated with oxidizing (hydrogen peroxide) and reducing (ascorbic acid) compounds at 37°C under aerobic conditions. The results showed that TiO_2_-NP generated relatively small amounts of acellular ROS when treated under both the oxidizing and reducing conditions (Fig. 6). Compared to that the Fe_2_O_3_-NP produced highest amount of ROS following the treatment of ascorbic acid and H_2_O_2_. To replicate the reducing conditions present within the cells, the NP were treated with whole cell lysate. Fe_2_O_3_-NP again generated high amounts of acellular ROS compared to TiO_2_-NP. These results demonstrate that Fe_2_O_3_-NP require reducing conditions to convert Fe(III) to Fe(II) before generation of ROS (Fig. 6).

### Intracellular ROS generation measured by H_2_DCFDA staining

**Figure 7:**
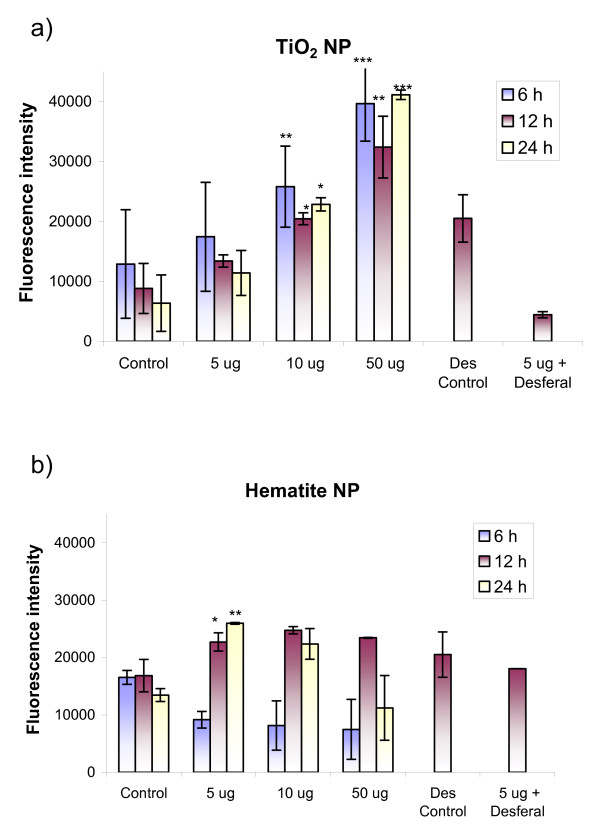
**Intracellular radical measurement after exposure of BEAS-2B cells to TiO_2_-NP (a) and hematite NP (b)**. Hematite NP induced a delayed effect (significant results after 12 h) compared to TiO_2 _NP (significant results after 6 h exposure). Desferal treatment (concentration: 100 μM) of particle-exposed cultures induced a significant reduction of radical formation in TiO_2 _but not in hematite exposed cells.

TiO_2 _nanoparticle exposure to the BEAS-2B cells prompted a concentration-dependent generation of intracellular ROS after 6 h exposure and was statistically significant at concentrations of 10 μg/cm^2 ^(p < 0.01) and 50 μg/cm^2 ^(p < 0.01) (Fig. 7a). Highest ROS production was observed at 50 μg/cm^2 ^for all exposure times. Parallel treatment of desferal (desferoxamine, 100 μM), an iron chelator and radical scavenger, along with the nanoparticles resulted in a reduction of free radical generation (Fig. 7a).

Hematite NP induced lower amounts of intracellular ROS compared to TiO_2_-NP. Also, the induced effect was delayed and statistically significant after 12 h exposure time. Desferal treatment did not reduce the amount of intracellular produced ROS significantly (Fig. 7b)

### 8-OHdG detection by ELISA technique

**Figure 8:**
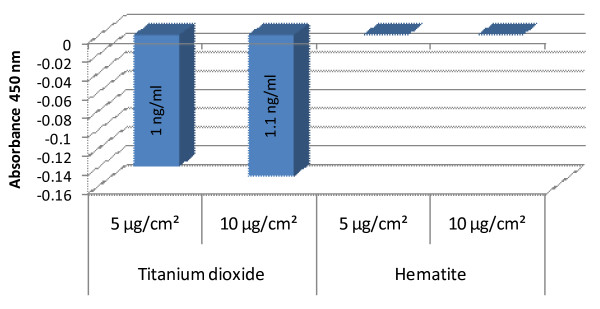
**Relative values of 8-OHdG adduct formation in IMR-90 cells after treatment with titanium dioxide and hematite nanoparticles for 24 h**. TiO_2_-NP were found to generate 1 and 1.1 ng/ml of 8-OHdG adducts at the concentrations of 5 and 10 μg/cm^2^, respectively, whereas hematite NP failed to induce any 8-OhdG adducts in IMR-90 cells.

For investigation of NP-induced oxidative DNA-damage the genomically unchanged IMR-90 cells were used. Exposure of IMR-90 cells to different concentrations of TiO_2_-NP for 24 h revealed that they were capable of inducing DNA adduct formation. Quantitatively, utilizing the pre-defined standards TiO_2_-NP were found to generate 1 and 1.1 ng/ml of 8-OHdG adducts at the concentrations of 5 and 10 μg/cm^2^, respectively, which was approximated to be in the whole genome of two million cells. (Fig. 8). Fe_2_O_3_-NP failed to induce any 8-OHdG-adduct formation in IMR-90 cells. The final results obtained from this study were negative values owing to the competitive type of ELISA.

## Discussion

The aim of this study was to investigate the toxicity of TiO_2_-NP and compare it to Fe_2_O_3_-NP. The factors taken into account for this study were: i) physico-chemical properties of the particles (size distribution, surface area, element analysis, zeta potential), ii) uptake and translocation of the particles by human cells, iii) capability of the particles to induce cyto- and genotoxicity in human lung cells, iv) primary (acellular) and secondary (intracellular) ROS generation, and v) genomic damage caused by oxidative stress (8-OHdG).

Analysis of the zeta potential of the NPs suspended in media without fetal bovine serum showed a high positive zeta potential for the TiO_2_-NP as compared to the negative zeta potential of the Fe_2_O_3_-NP. Similar studies with same observations were carried out by de la Garza *et al. *[8] and Wang *et al. *[9]. These differences in zeta potential of TiO_2 _and Fe_2_O_3_-NP can influence the particle uptake as well as toxicological parameters like ROS generation and genomic damage.

The results obtained in this study are summarized in Tab. 1 and demonstrate the different toxicological properties of both types of NP.

**Table 1:** Summary of the observed effects caused by TiO_2_- and hematite-NP

	**TiO_2_-NP**	**Hematite-NP**
Genotoxicity (Comet assay: DNA breakage)	-	+

Cytotoxicity (Trypan blue assay)	- (+)* ^1^	+

Acellular radical formation (ESR)	↑	↓ (↑)*^2^

Intracellular radical formation (H_2_DCFDA)	↑	delayed

Oxidative DNA-damage (8-OHdG)	+	-

*1 cytotoxicity negative in BEAS-2B and positive in IMR-90 cells

*2 elevated radical formation under reducing conditions

The TEM study with TiO_2_- and hematite-NP showed that both types of NP were actively taken up by human lung cells. We observed a filopodia assisted engulfment of the agglomerats of nanoparticles. Filopodia are slender cytoplasmic projections present at several cell types (including lung epithelial cells), which extend from the leading edge of migrating cells. These appendages had been previously found to form points of adhesions by the cell to surface [10]. They also help in cell to cell attachment and signalling. Recently, they had been found to be involved in assisting the cells by acting as phagocytic tentacles [11]. Stearns *et al. *[12] demonstrated the uptake of TiO_2 _nanoparticles in clusters by lung epithelial cells (A549) using transmission electron microscopy.

Following uptake and intracellular translocation all the particles were found near to the peri-nuclear space. Recently, similar to our uptake findings Park *et al. *[13] had demonstrated the translocation of cerium oxide nanoparticles to the peri-nuclear region as agglomerated particles followed by their penetration into the cytoplasm directly and within vesicles. Unlike the finding of Chen and von Mikecz [14] and Li *et al. *[15] demonstrating penetration of nucleus and mitochondria by SiO_2 _and ambient air nanoparticles, we found no nanoparticle penetrating these organelles in our study.

While, TiO_2_-NP did not cause any cytotoxicity in lung epithelial (BEAS-2B) cells, it caused significant loss of cell viability in lung fibroblasts (IMR-90), especially at longer exposure times. However, these particles were found to be non-genotoxic in both the cell lines using the Comet-Assay which detects DNA-breakage. Previously, we had found that the native titanium dioxide (anatase) nanoparticles were non-cyto- and -genotoxic in V79 cells (Chinese hamster lung fibroblasts) [16]. For the genotoxicity study in these cells the micronucleus assay was used which detects both chromosome breakage as well as aneuploidy.

In contrast to our study Turkez *et al. *[17] demonstrated that TiO_2 _nanoparticles were capable of causing genotoxicity by inducing sister chromatid exchange and micronucleus formation in human white blood cells. Recently, Thevenot *et al. *[18] had also demonstrated that the surface chemistry of the TiO_2 _nanoparticles play a major role in increasing or decreasing its toxicity.

In contrast to TiO_2_-NP, the iron-containing NP hematite induced a significant amount of DNA-breakage in human lung cells. The magnitude of genotoxic effect observed in IMR-90 cells at the time point of 24 h and a concentration of 50 μg/cm^2 ^was five fold higher compared to BEAS-2B cells. IMR-90 cells are non-transformed "normal" human lung fibroblasts which are more sensitive after particle exposure than the SV-40 transformed immortalized human bronchial epithelial cells BEAS-2B.

Earlier research done with hematite fine particles demonstrated them to be non-genotoxic in different cell lines and reacted intracellularly only by enhancing the effects of other toxic substances such as benzo [a]pyrene [19,20]. In own studies with fine hematite particles (∅ ≤ 2.5 μm) we observed a lower genotoxic response compared to ultrafine hematite particles [21]. Therefore, we suggest that Fe_2_O_3_-NP react quite differently as compared to their finer counterparts. Also, we conclude from our study that NP do not directly interact with DNA (indirect genotoxic effects).

Analysis of acellular ROS generation capability of the nanoparticles showed that TiO_2 _as well as hematite -NP induced elevated amounts of acellular ROS. Under oxidizing and reducing conditions hematite NP induced significant acellular ROS generation in contrast to TiO_2_. The results of the EPR-experiments with TiO_2_-NP are in agreement with those of Braydich-Stolle *et al. *[22], who have stated that under normal conditions TiO_2_-NP were capable of generating just small quantities of acellular ROS. They have also observed that the nanoparticles form aggregates when dispersed in water and serum free media with the largest aggregates found in the serum free media.

However, if hematite-NP were pre-treated with ascorbic acid or whole cell lysate they generated the highest amount of OH• radicals independent of the time points and the applied concentration. The results pointed towards the property of agglomeration by the nanoparticles along with the alteration of surface ions from Fe(III) to Fe(II). He *et al. *[23] have demonstrated that smaller particles in the nanosize range tend to agglomerate when suspended in a low pH liquid. Therefore, the increase in the size of agglomerates at lower pH was directly proportional to the size of the particles. Therefore, following the conclusions of He *et al. *[23] it may be presumed that the Fe_2_O_3_-NP after reacting with the reducing agent (ascorbic acid) had an alteration of their surface ionic composition and surrounding pH. This might cause the nanoparticles to separate from their agglomerates and produce an increased surface area and highest generation of acellular ROS. Also, Fe(II) can lead to direct reduction of hydrogen peroxide to OH• radicals following the Fenton reaction.

Intracellulary, significant amounts of ROS were generated by TiO_2_nanoparticles in dependence upon particle concentration. TiO_2 _is known to have a photocatalytic activity. This might be a reason for the higher intracellular ROS generation compared to hematite-NP. Co-treatment of TiO_2 _particles with desferal reduced the ROS generation after a short time exposure of 12 h. Intracellular ROS formation caused by hematite-NP was delayed compared to TiO_2_-NP. The above discussed chemical reactions at the particle surface might be responsible for this effect.

In our study we further analysed the formation of 8-OHdG adducts in NP-exposed IMR-90 cells. Interestingly, eventhough the TiO_2_-NP were non-genotoxic using the Comet-assay, they generated high amounts of 8-OHdG probably caused by the observed high intracellular ROS generation. Gurr *et al. *[24] have shown that TiO_2 _-NP induce mainly generation of hydrogen peroxide and nitric oxide leading to lipid peroxidation and oxidative DNA damage in lung epithelial cells. Kang *et al. *[25] had observed an activation of DNA damage check-points and an up-regulation of P53 along with DNA damages caused by ROS generation by these nanoparticles in peripheral blood lymphocytes. No 8-OHdG adduct formation could be observed following Fe_2_O_3_-NP exposure. Previously, 8-OHdG DNA adduct formation by hematite particles was only observed when the particles were coated with B(a)P [26].

From the present study it can be concluded that even though the TiO_2_-NP demonstrated low amount of cyto- and genotoxicity as compared to the Fe_2_O_3_-NP, they were more capable of generating stable 8-OHdG adducts. This may be related to the surface charge of the NP. The study also demonstrated that the Fe_2_O_3_-NP required a special reducing condition to undergo conversion from Fe(III) to Fe(II) according to the Fenton reaction to generate OH radicals. Generation of intracellular reactive oxygen species by TiO_2_-NP may induce the observed oxidative DNA adduct formation. Even low adduct levels may lead to persistent DNA lesions. Other factors that need to be considered are potential DNA repair inhibitions caused by TiO_2_-NP. Further studies are needed to eludicate the mechanisms behind the observed effects.

## Materials and methods

### Particle source

TiO_2_-NP (∅ < 100 nm) were obtained in powder form from Degussa GmbH, Germany. The Fe_2_O_3_-NP (∅ < 100 nm) were bought from Sigma-Aldrich (Cat No.- 544884). The surface area of the particles was measured using the Brunauer-Emmett-Teller (BET) technique and was performed at the Nanolab, FOCAS Institute, Dublin, Ireland. For BET the samples were analyzed in dry state by nitrogen adsorption after degassing at 300°C (Micrometrics Gemini 2360). The surface area of the TiO_2_-NP was 49.71 ± 0.19 m^2^/g. Fe_2_O_3_-NP were found to have a surface area of 34.39 ± 0.17 m^2^/g. Zeta potential following Smoluchowski model and particle size distribution was measured using dynamic light scattering (dls) technique on Malvern's nano zetasizer ZS series in serum free liquid media.

Determination of the trace iron elements present on the particles was done using 1,10 phenanthroline chloride dye and spectrophotometric technique following the protocol described by Pyenson and Tracy [27] with minor modifications specified by German Industrial norms (DIN: 38 406). In brief 70 mg of each was suspended in 20 ml of H_2_O. Five ml of aqua regia were added to the suspension and heated at 70 – 90°C. The solution was evaporated till only 2 ml of final volume were left. Two ml of concentrated H_2_SO_4 _were added to this mixture to convert all of the Fe present to Fe(III). Conversion of the solution to a milky white colour was observed. Heating was further continued till the suspension turned to creamy white. Twenty ml of H_2_O were added to this precipitate and stirred till a clear solution was obtained. The solution was diluted to a final amount of 0.714 mg/100 ml. It was filtered through a 0.45 micron filter and to 50 ml of this solution 40 ml of ammonium acetate (buffer) were added. Two millilitres of hydroxylammonium chloride, each followed by 2 ml of 1,10-phenanthroline chloride, were added to the solution while stirring. After 15 min the absorbance of the final solution was measured at 510 nm. Another, 50 mg of the particles were suspended in 100 ml of MilliQ H_2_O and stirred for 1 h under anaerobic condition. After 1 h the suspension was filtered through a 0.45-micron filter. Twenty millilitres of filtrate were taken and mixed with 5 ml of ammonium acetate. While stirring 2 ml of hydroxylammonium chloride were added to the solution along with 2 ml of 1,10-phenanthroline chloride. This mixture was still for 15 min, and then the absorption spectra were measured at 510 nm. The obtained spectroscopic values of the latter were subtracted from the former value to obtain the absolute value of Fe(III) and determine any Fe(II) present on the particle surface. While, TiO_2 _-NP were found to be absolutely pure in their composition, Fe_2_O_3 _– NP contained only Fe(III) without any tracer amount of Fe(II). Quantitatively, the amount of Fe(III) present in 0.4 mg of Fe_2_O_3_-NP was equivalent to 935 μM.

### Cell culture

Human diploid fibroblasts (IMR-90) were obtained from the American type culture collection (ATCC, CCL-186). The cells were grown in Earl's Modified Eagle's Medium (EMEM) supplemented with 2 mM glutamine, 1% non-essential amino acids, 10% foetal bovine serum (FBS) and 0.5% gentamycin. The doubling time of IMR-90 was about 48 h. The cultures were maintained at 37°C and 5% CO_2_.

SV-40 virus-transformed human bronchial epithelial cells (BEAS-2B) were obtained from the European collection of cell cultures (ECCC, 95102433). The cells were grown in defined keratinocyte serum free medium (D-KSFM) supplemented with special epithelial cells growth supplements (1 ml) and 0.5% gentamycin. The cultures were maintained also at 37°C and 5% CO_2_.

### Scanning Electron Microscopy (SEM)

Dry particle samples (TiO_2_-NP, Fe_2_O_3_-NP) were mounted on the stage of the SEM (DSM 960 A, Zeiss Oberkochen, Germany) and treated with a high-energy electron beam. The resulting X-rays emitted from the samples and images acquired as well as the morphology of particles were analysed.

### Transmission electron microscopy (TEM)

At the end of incubation time, cells were washed thrice with serum free medium and fixed with 4% glutaldehyde in 0.1 M sodium phosphate buffer pH 7.4 for 1 h at 4°C. After postfixation in 1% OsO_4 _in 0.1 sodium phosphate buffer for 1 h at 4°C, the samples were washed in PBS, dehydrated in a graded series of acetone, and embedded in the epoxy resign Araldite (Fluca Buchs, Germany). Ultrathin sections were cut with the Ultramicrotome Ultracut S (Leica), mounted on copper grids, stained with uranyl acetate and lead citrate and studied in a transmission electron microscope (TEM) EM 902 A with EELS or Libra 120 with EELS and EDX (Zeiss Oberkochen, Germany). Digital pictures were captured using a CCD camera 2 K (Proscan).

### Trypan blue assay

For the cytotoxicity analysis approximately, one million cells were plated and after 24 h pre-incubation cells were exposed to the different concentrations of particles for different time periods. After elapse of the exposure time the media were removed. The cells were washed twice with 2 ml of pre-warmed PBS and then trypsinized. The cells were then resuspended in 300 μl of PBS mixed with Trypan blue (0.5%) in a 1:1 ratio and kept in an incubator for 4 min at 37°C. Cells were then counted with a haemocytometer, and the cell viability was calculated.

All the experiments were conducted in triplicates and the statistical significance was analyzed with one-way ANOVA, using the Holm-Sidak method and pair-wise analysis.

### Alkaline comet assay

The experiment for detection of genotoxicity was performed with one million cells which were exposed to different particle concentrations for different time intervals. After lapse of the exposure time, cells were washed, trypsinized and suspended in low melting agarose and cased onto a gel bond film. After the agarose was solidified it was suspended in freshly prepared and pre-cooled cell lysis buffer overnight. The following day, electrophoresis was conducted in alkaline electrophoresis buffer for 10 mins (conditions: 300 mA, 1.5 V/cm at 4°C). After completion of the electrophoresis run time, the Gelbond™ film was treated with neutralisation solution for 30 min and then dehydrated in absolute CH_3_COOH for 2 h. Gels were stored in the dark overnight at 4°C to let them dry completely until stained with SYBR-Green nucleic acid stain. Image analysis was performed using 'Comet Assay IV' software (Perspective Instruments, UK) and Leica microscope attached to a CCD camera. Values of the olive tail moment (OTM) were automatically calculated by the software. All experiments were carried out twice in duplicate, and the statistical analysis was done using Student's t-test.

### Electron paramagnetic resonance (EPR)

The EPR spectroscopy technique is used for studying chemical species having one or more unpaired electrons and a short life-span, such as free radicals or inorganic complexes possessing a transition metal ion. The basic physical concepts of EPR are based on the fact that an electron is a charged particle, which spins around its axis causing it to act like a tiny bar magnet. Technically, this property of the electron is known as a magnetic moment. The EPR measures this spin of the electrons using a spin trap such as 5, 5-dimethyl-1-pyrroline-N-oxide (DMPO), which forms a stable product (spin adduct) after reacting with the free radical.

DMPO reacts directly with the hydroxyl radicals (OH) forming the product DMPO-OH or the decomposition of DMPO-OOH, having a half-life of approximately 1 min in neutral media.

The generation of the hydroxyl radical by TiO_2 _particles at concentrations of 10 μg/cm^2 ^and 50 μg/cm^2 ^was measured with EPR under the following conditions:

#### Treatment of particles with H_2_O_2_

The particles were suspended at the concentration of 50 μg/cm^2 ^in double distilled water (DdH_2_O) and treated with 50 μl of 1 M DMPO spin trap. DdH_2_O was used as negative control. The suspension was incubated for 1 h at 37°C and filtered through a 0.45 μm Acrodisc syringe filter. The filtrate was immediately transferred to a capillary and measured with an EPR spectrometer.

#### Treatment of particles with ascorbic acid and H_2_O_2_

The particles were suspended in a petri dish at concentrations of 10 and 50 μg/cm^2^, respectively, in the presence of ascorbic acid (2 μg/ml) followed by the addition of 50 μl of 1 M DMPO spin trap. DdH_2_O was used as negative control again. The suspension was incubated for 1 h at 37°C and filtered through a 0.45 μm Acrodisc syringe filter. The filtrate was immediately transferred to a capillary and measured with an EPR spectrometer.

#### Treatment of particles with whole cell extract (WCE) and H_2_O_2_

The WCE was prepared with BEAS-2B cells grown in 175 cm^2 ^flask following the protocol of Wang *et al. *[28]. Approximately, 100 million cells were taken for the preparation of the WCE. The cells were trypsinized, collected and washed with cold PBS one time. They were centrifuged at 1500 rpm for 5 min at 4°C. The cell pellet was then washed and suspended in a hypotonic buffer. The cells were freeze-fractured by quick freezing at -80°C and thawing at 37°C. Subsequently, the mixture was incubated at 4°C for 30 min on a rotating platform in KCl at a concentration of 500 mM and then centrifuged (14,000 rpm) at 4°C for 40 min, then the supernatant was removed and frozen at -20°C.

Final concentration of the protein in WCE was measured using the Bradford method (Dye – Coomassie Blue G-250 in acidic solution) and following the manufacturer's protocol (Bio-Rad Protein Assay). Briefly, protein standards of 500 μg/ml, 400 μg/ml, 300 μg/ml, 250 μg/ml, 200 μg/ml, 100 μg/ml and 50 μg/ml were prepared. Ten microlitres of these standards and the probes were mixed with 200 μl of the dye reagent and mixed for 15 min at room temperature. Measurement was taken using a plate reader at 570 nm absorbance. A standard curve was drawn from the standard measurements using which the amount of protein in the WCE was determined to be approximately 450 μg/ml.

The particles were suspended in a Petri dish at concentrations of 10 and 50 μg/cm^2^, respectively along with 250 μl of WCE followed by the addition of 50 μl of 1 M DMPO as spin trap and 120 mM H_2_O_2_. DdH_2_O was used as negative control. The suspension was incubated for 1 h at 37°C in a CO_2 _incubator and the suspension was filtered through a 0.45 μm Acrodisc syringe filter. The filtrate was immediately transferred to a capillary and measured with an EPR spectrometer.

All the EPR spectra measurements were recorded at RT and the instrument was set to: magnetic field 3360 G, sweep width 100 G, scan time 30 sec, modulation amplitude 1975 G, receiver gain 1000. Quantification was done by accumulation of three different spectra each averaging three different scans. All four peaks were the amplitude. Outcomes were expressed as the total amplitude in arbitrary units (ESR units; ESR = electron spin resonance).

### 2',7'-Dichlorodihydrofluorescein diacetate (H_2_DCFDA)

H_2_DCFDA is a fluorogenic probe commonly used to detect intracellular generation of reactive oxygen species (Invitrogen, Eugene, Oregon, USA).

For the experiments one million cells were plated in 25 cm^2 ^flasks and pre-incubated for 24 h. The old media was then replaced by a fresh one and the cells were exposed to the particles at different concentrations for different time intervals. After the lapse of exposure time cells were washed and suspended in 3 ml of PBS. They were exposed to a working solution of H_2_DCFDA at the final concentration of 10 μM for 20 min and incubated at 37°C. The PBS and the dye were then removed, and the cells were trypsinized with a low concentration of trypsin (0.05%) and reincubated for 10 min. Finally, 200 μl of PBS/HBSS were transferred into the culture flask and the cells were resuspended in it. This suspension was taken into a 96-well plate and immediately measured with an enzyme-linked immunosorbent assay (ELISA) plate reader set at an excitation wavelength of 485 nm and an emission wavelength of 535 nm.

All experiments were performed twice in duplicates, and for statistical analysis Student's t-test was applied.

### 8-Hydroxyl-2-deoxyguanosine (8-OHdG) detection by the enzyme-linked immunosorbent assay (ELISA) technique

Free radicals have an affinity to damage the DNA bases leading to their modifications. Of these, 8-OHdG with a hydroxyl group at the eight position of guanine is formed easily and abundantly by oxidative stress [29]. The protocol used for the isolation of DNA from the IMR-90 cells without the formation of 8-OHdG adducts was adopted from the work of Helbock *et al. *[30].

For the measurement of 8-OHdG using an ELISA kit 200 μg of DNA extract were suspended in 135 μl of H_2_O. Fifteen microlitres of 200 mM sodium iodide and 6 units of nuclease-P1 were then added to the DNA solution and incubated for 30 min to 1 h at 37°C after argon substitution.

Fifteen microlitres of 1 M Tris-HCl buffer (pH 7.4) and 2 units of alkaline phosphatase were added to the solution and incubated for 30 min – 1 h at 37°C after argon substitution. To remove all macromolecules and enzymes the hydrolysates were filtered through Millipore microcon YM-10 at 14000 rpm for 10 min.

All the buffers were brought to room temperature. Fifty microlitres of the standards (0.5, 2, 8, 20, 80, and 200 ng/ml) and the DNA samples of the exposed cells were applied onto the wells. The 50 μl primary antibody was applied to the wells followed by shaking and kept overnight (4°C) after sealing it with an adhesive strip. On the next day the solution in the well was thrown away followed by removal of the residue by tapping onto absorbent towels several times. The assay plates were then washed with 250 μl of wash buffer 3 times. One hundred microlitre of the secondary antibody was then added to the wells and the assay plate was sealed tightly with an adhesive tape and incubated for 1 h (room temperature). After the lapse of time the washing steps followed to remove the excess primary antibodies was followed. One hundred microlitre of chromatic solution (3,3,5,5-tetramethyl-benzidine) were added to the wells followed by incubation for 15 min RT in dark. The chromatic solution had changed to blue colour. The reaction was stopped by adding 100 μl of a reaction terminating solution (2 M H_2_SO_4_) to the wells, and the absorbance was measured at 450 nm (reference wavelength – 620 nm). The standard curve was used to determine the amount of 8-OHdG present in the samples.

## Competing interests

The authors declare that they have no competing interests.

## Authors' contributions

All authors have read and approved the final manuscript. KB did the experimental work related to and wrote the research article together with ED. MD provided the facilities for performing the physico-chemical analysis of the nanoparticles and advise related to the analysis of the data. JB did the spectroscopic iron determination experiment along with KB. RS provided the facilities and advice related to the electron spin resonance technique for the measurement of the acellular reactive oxygen species. EH performed the electron microscopy studies. ED provided funding for the experiments and supervised the entire work.
